# Robot-Assisted Percutaneous Balloon Compression for Trigeminal Neuralgia: Technique Description and Short-Term Clinical Results

**DOI:** 10.3389/fsurg.2022.869223

**Published:** 2022-03-18

**Authors:** Qiangqiang Liu, Junjie Wang, Changquan Wang, Wenze Chen, Wenzhen Chen, Xiaolai Ye, Ziyu Mao, Chencheng Zhang, Jiwen Xu

**Affiliations:** ^1^Department of Neurosurgery, Clinical Neuroscience Center Comprehensive Epilepsy Unit, Ruijin Hospital, Shanghai Jiao Tong University School of Medicine, Shanghai, China; ^2^Clinical Neuroscience Center, Ruijin Hospital Luwan Branch, Shanghai Jiao Tong University School of Medicine, Shanghai, China; ^3^Department of Neurosurgery, Center for Functional Neurosurgery, Ruijin Hospital, Shanghai Jiao Tong University School of Medicine, Shanghai, China; ^4^Shanghai Research Center for Brain Science and Brain-Inspired Technology, Shanghai, China

**Keywords:** trigeminal neuralgia, percutaneous balloon compression, robotics, stereotactic neurosurgery, technique

## Abstract

**Objective:**

Percutaneous balloon compression (PBC) is a minimally invasive treatment for trigeminal neuralgia (TG) with a favorable cost-effectiveness ratio, but this technique has a steep learning curve. This study presents our initial clinical experience of robot-assisted PBC using a neurosurgical robot on six consecutive patients with TG.

**Methods:**

We fixed the patient's head with a skull clamp and connected it with the linkage arms of a Sinovation^®^ neurosurgical robot, which was then registered using four bone fiducials by the robotic pointer. The puncture needle was positioned at the entry point on the skin using a robotic arm and advanced to the target point after the skin had been incised with a pointed surgical blade. This procedure was repeated for a second trajectory. A balloon was then advanced and inflated using 0.3 ml of a contrast agent. Upon injection of 0.6 ml contrast agent, the ganglion was kept compressed for 120 s. After removal of the balloon and puncture needle, compression of the face was performed to achieve hemostasis.

**Results:**

All patients achieved immediate pain relief following PBC. No permanent or severe complications were registered, and there was no pain recurrence in any of the patients during the follow-up period.

**Conclusions:**

Despite requiring a longer time for preoperative preparation, robot-assisted PBC provided a high degree of accuracy and safety, and it can also shorten the learning curve for surgeons unfamiliar with PBC. Robot-assisted surgical approaches should be further developed and adopted for PBC.

## Introduction

Trigeminal neuralgia (TG) is a facial pain disorder characterized by sharp and paroxysmal pain confined to areas innervated by the trigeminal nerve (1, 2). Because of the sudden and stabbing-like episodic pain that characterizes it, TG negatively affects patients' quality of life (1). Microvascular decompression (MVD) is recommended as the first therapeutic option because it provides pain relief for longer duration, but the fact that it involves major intracranial surgery makes it unsuitable for elderly or infirm patients (3). In addition, some patients are unwilling to undergo MVD or are unresponsive to surgical treatment. Alternative treatment options include percutaneous approaches such as percutaneous balloon compression (PBC), glycerol rhizolysis (GR), and radiofrequency ablation (RF) (4–7).

PBC of the gasserian ganglion, first described in 1983 by Mullan and Lichtor (8) is a minimally invasive treatment for TG that has recently regained popularity and attention because of its favorable cost-effectiveness.

Although PBC and other percutaneous procedures can be easily performed by experienced neurosurgeons with good results and considerably low complication rates, mastering of the technique involves a steep learning curve. Additionally, serious complications associated with PBC leading to severe morbidity and death have been reported (9). These complications are mostly related to needle misplacement in different foramina, incorrect foramen ovale puncture, repeated attempts at needle placement, or the use of a cannula of the wrong size. The identification of the foramen ovale using conventional monoplanar fluoroscopy may not always be adequate. In addition, natural anatomical variations encountered in 2–4% of patients may prevent the puncturing of the foramen (10). Several methods have been proposed to improve the success rate of the foramen ovale puncture, including intraoperative C-Arm, 3D-computed tomography (CT), neuronavigation, and personalized 3D-printed jig plates (11–13).

This study presents our initial clinical experience of robot-assisted PBC using a Sinovation^®^ neurosurgical robot (Sinovation, Beijing, China).

## Materials and Methods

### Study Population

Our study cohort consisted of six consecutive patients with TG who underwent PBC at the Clinical Neuroscience Center of the Ruijin Hospital in China between May and August 2021. All the participants provided their informed consent for inclusion in the study. TG diagnosis was verified using standard clinical criteria (presence of trigger points, neurologic examination of cranial nerve V function and presence of paroxysmal pain). Patients were also screened for drug resistance, defined as a failure in controlling pain using carbamazepine at a maximum dosage of 1,200 mg per day for 6 months.

### Preoperative Preparations

All patients underwent magnetic resonance scanning (United, uMR 890), which consisted of T1 magnetization-prepared rapid acquisition gradient echo, T2-weighted fluid-attenuated inversion recovery, and thin-slice head CT scanning.

We utilized stainless steel self-tapping bone-screws (Sinovation, Beijing, China) as bone fiducials. On the morning of surgery, five fiducials were screwed into the skull using a battery-powered auto driver after sterilization and infiltration of local anesthetic. We utilized the four bone fiducials for robot registration and another bone fiducial for verification. Head CT scans with 1-mm slice thicknesses, without intervals, were obtained.

### Surgical Plan

**Figure 1:**
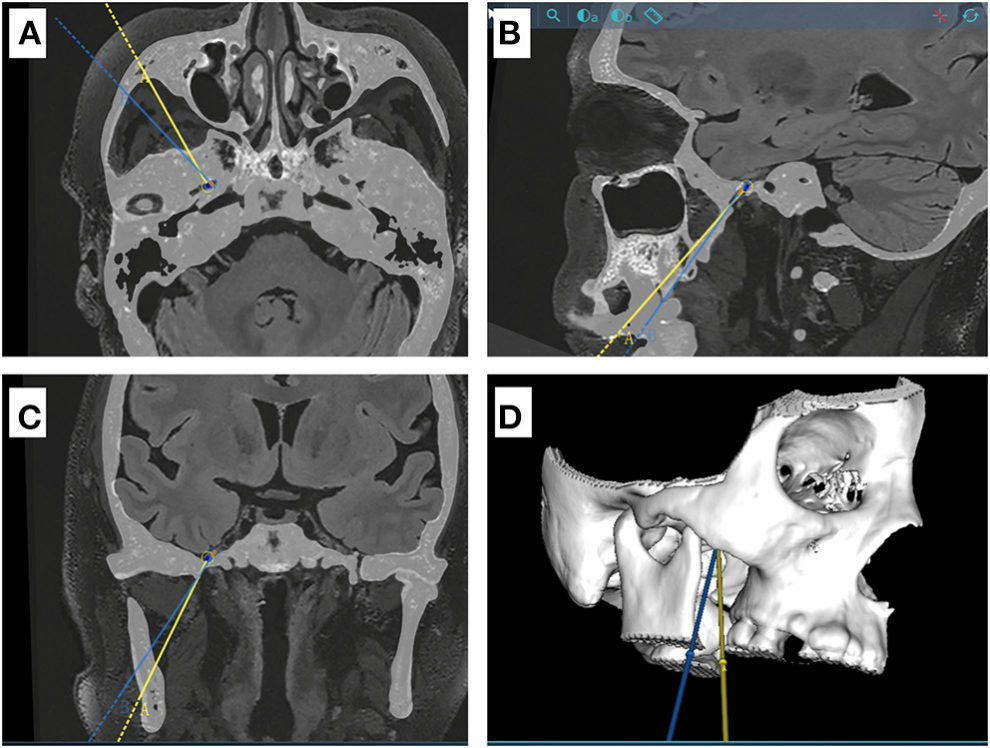
Schematic diagram for two different puncture trajectories in a sample case. **(A–C)** CT and MR Flair sequence fusion images showing the projections of the two puncture trajectory A and B (yellow and blue line, respectively) in axial, sagittal and coronal positions. Trajectory B is located outside and below trajectory A. **(D)** three-dimensional model of the skull base.

**Figure 2:**
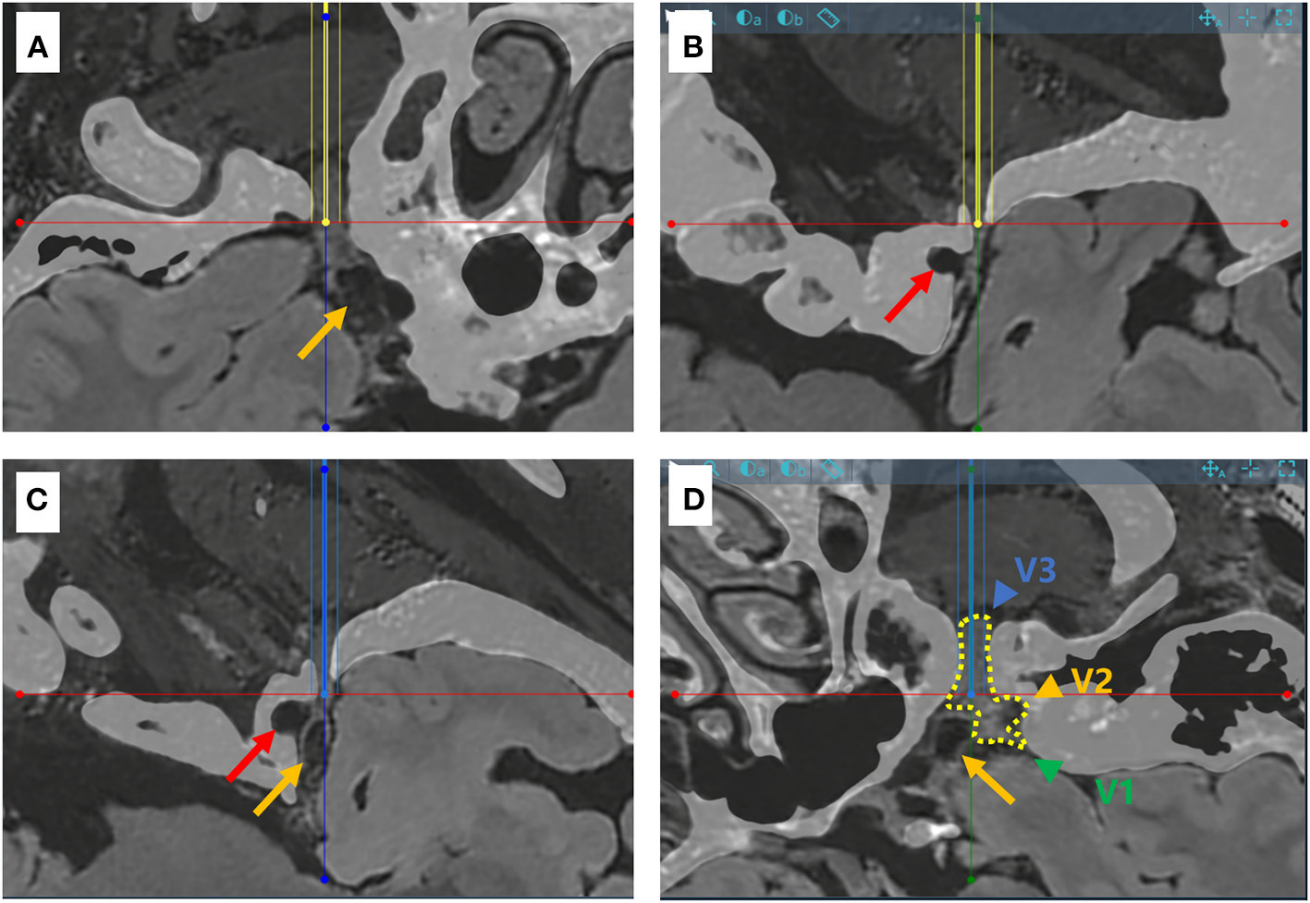
Schematic diagram for the puncture targets of two different trajectories in a sample case. **(A,B)** CT and MR flair sequence fusion images showing the needle-path perspective for trajectory A (yellow line). The trajectory penetrates the foramen ovale smoothly, but not Meckel's cave (yellow arrow). The red arrow points to the internal carotid artery. **(C,D)** Needle-path perspectives for trajectory B (blue line), which effectively reaches Meckel's cave. The dotted line in yellow shows the three branches of the trigeminal nerve (V1, V2 and V3, green, represented by yellow and blue arrows respectively).

**Figure 3:**
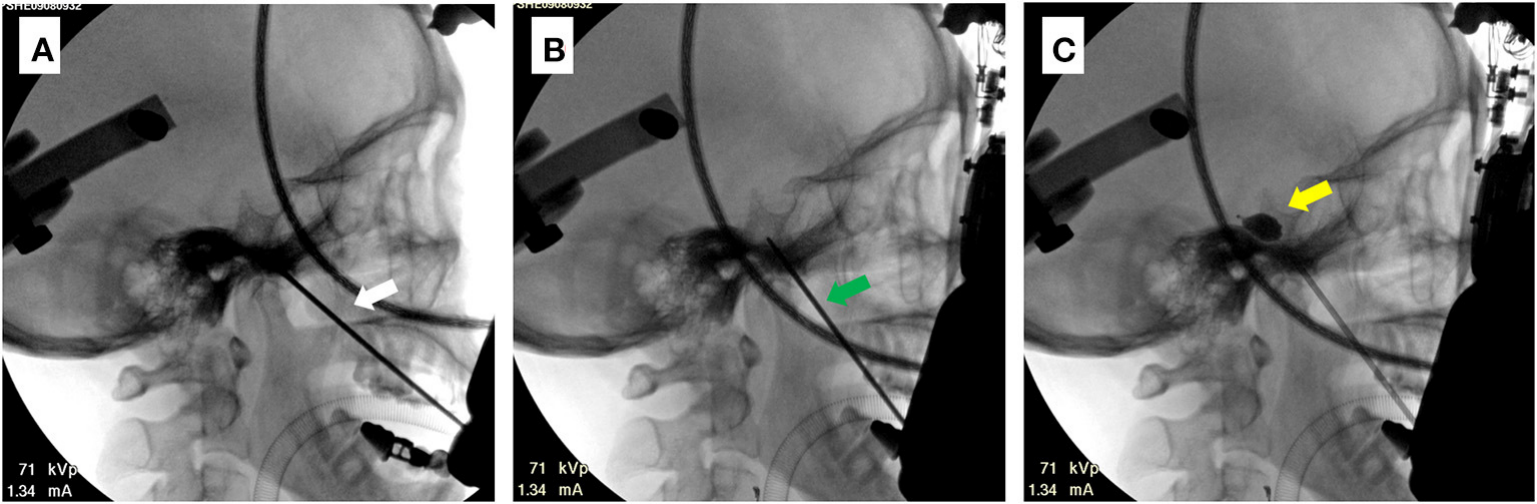
Intraoperative C-arm images for a sample case (patient 6). **(A)** Image of completed trajectory A (white arrow). **(B)** Image of completed trajectory B (green arrow). The direction of trajectory can be observed to change. **(C)** Image of the balloon after injection of the contrast agent. The inflated balloon can be distinguished by its piriform appearance (yellow arrow).

All image data were transferred to a Sinovation planning station (version 2.0.1.2; a portable computer; Sinovation, Beijing, China). Our Surgical plan included two trajectories. In trajectory A, the entry point was set on the face (2.5 cm lateral to the angle of the mouth), and the target point was set on the foramen ovale. In trajectory B, the target point was set on Meckel's cave, and the entry point was set based on the foramen ovale and Meckel's cave (Figure 1). The two trajectories would be optimized on the 3D view of the skull and the trajectory view based on the preoperative MR and CT images (Figure 2). A C-arm monoplanar fluoroscopy machine (GE, Wisconsin, USA) was set only for lateral imaging to check the correct position of the cannula and the balloon shape (Figure 3).

### Robot-Assisted Percutaneous Balloon Compression

The surgery was performed under general anesthesia. The patient was placed in the supine position with a slight neck extension. We used the DORO QR3 skull clamp with three head fixation points to fix the patient's head and confirm that the centerline of the head is perpendicular to the C-arm monoplanar fluoroscopy machine. The skull clamp was then connected with the linkage arms of the Sinovation^®^ neurosurgical robot. The surgical plan was uploaded to the Sinovation^®^ neurosurgical robot. The Sinovation^®^ neurosurgical robot was then registered using these four bone fiducials by the robotic pointer. The registration process was repeated if the registration error was above 0.3 mm. The robotic pointer was then utilized to touch the center of the fifth bone fiducial for visual verification of the registration error. Therefore, only registration errors below 0.3 mm were accepted.

**Figure 4:**
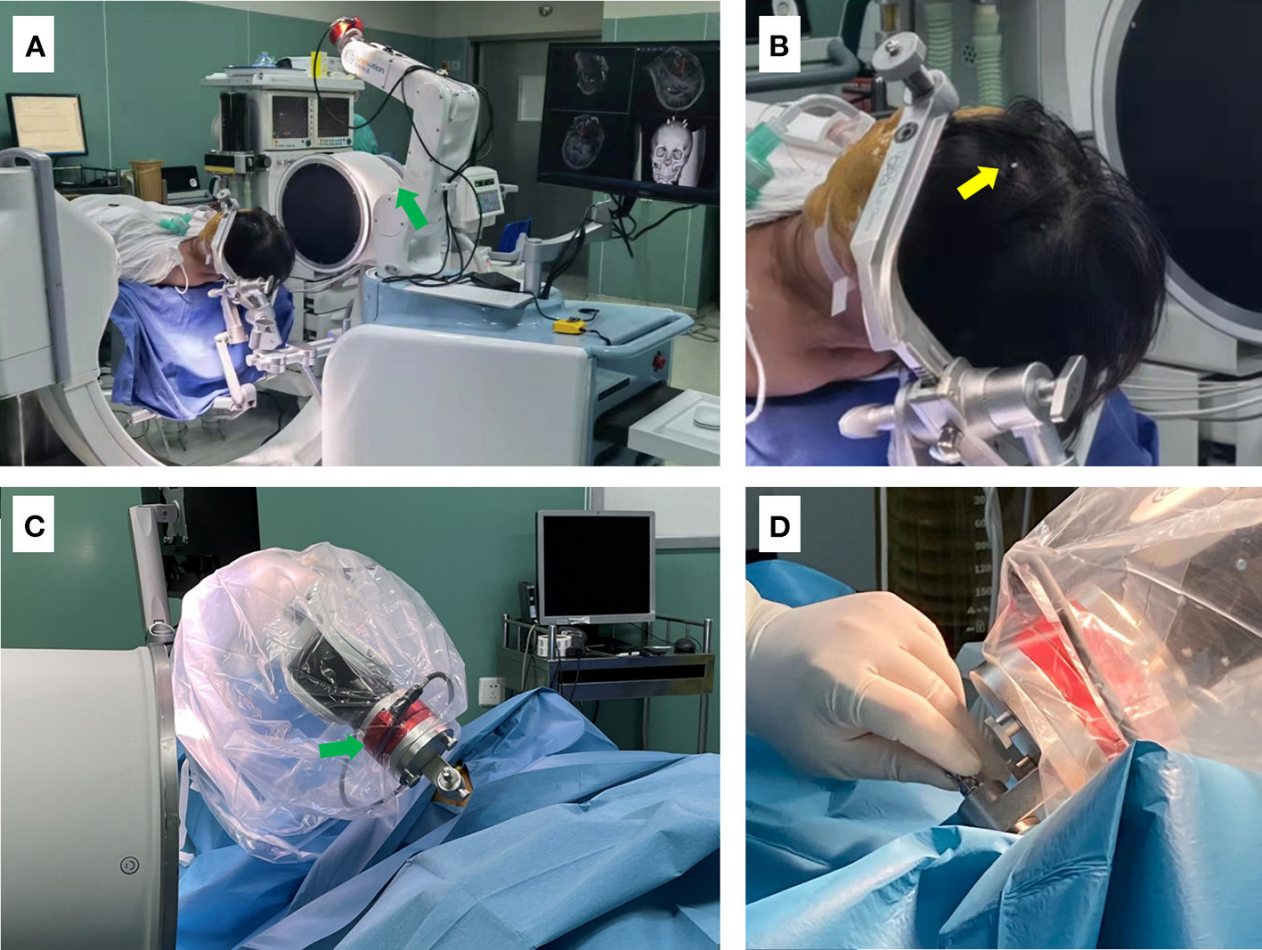
Photographs depicting the setup for the surgical procedure. **(A)** The patient is shown placed in supine position after general anesthesia, with the head fixed using a DORO head frame. The C-arm was then placed in position, and the head frame was connected and fixed to the robot. **(B)** Enlarged view showing the position of bone markers (yellow arrow). **(C,D)** The robotic arm of the surgical robot (green arrow) is shown guiding and supporting the puncture needle.

Fourteen gauge puncture needles were chosen to exactly fit the size of the robotic holder (inner diameter 2.2 mm). The puncture needle was positioned at the entry point on the skin using a robotic arm. The puncture needle was advanced to the target point after the skin was incised with a pointed surgical blade. The location of the puncture needle was confirmed using an X-ray machine. The robotic arm was then adjusted to follow the second trajectory, and the puncture needle was again advanced to the target point. A fine puncture needle was then advanced, with its position confirmed by X-rays. Lidocaine was injected into the trocar to reduce cardiac responses. A balloon was then advanced, with the position of its tip confirmed by X-rays. After the balloon was inflated with a 0.3 ml contrast agent, its shape was checked. Upon injection of a total of 0.6 ml contrast agent, the balloon ended up adopting its piriform appearance. Subsequently, the ganglion was kept compressed for 120 s. After this, the balloon and the puncture needle were removed. Compression of the face was performed to achieve hemostasis, and the surgery was finally completed (Figure 4).

Patient data (age, sex, presence or absence of apparent neurovascular conflict, neurologic status before and after surgery, and preoperative and postoperative incidence of herpes labialis) were recorded, along with the number of puncture attempts, the number of adjustments, and surgery duration.

### Efficacy Evaluation

The postoperative follow-up was completed by nonsurgical doctors monthly, via telephone, text messages, outpatient service, or mail. Postoperative facial pain was measured using the Barrow Neurological Institute (BNI) Pain Intensity Score (14), and facial numbness was determined using the BNI facial numbness score (15).

## Results

### Patient Data

A total of six consecutive patients with TG underwent frameless robot-assisted PBC at the Ruijin Hospital Luwan Branch from May to August 2021. All procedures were performed by a single surgeon (Q.Q. LIU). The mean age of the patients at surgery was 65 years (range: 59–75 years). The symptomatic period before undergoing PBC ranged from 2 to 15 years. Two patients had already undergone previous MVD surgery, and one patient had already undergone radiofrequency ablation. There were no postoperative complications such as bleeding or infection. The mean follow-up time was 4.5 (range: 3–6) months. A summary of the patients' characteristics and surgery results is presented in Table 1.

**Table 1:** Summary of the patients' characteristics and surgery results.

**No**.	**Gender**	**Age (years)**	**Symptoms' duration (years)**	**Side**	**Distribution of symptoms**	**Previous surgery**	**BNI Pain Intensity Score**	**BNI facial numbness score**	**Other complications**	**Registration error (mm)**
1	Female	59	8	Left	V3	MVD	I	II	-	0.17
2	Male	70	6	Right	V2-3	-	I	II	Facial muscles weakness	0.13
3	Female	64	15	Right	V2-3	MVD	I	III	-	0.14
4	Male	75	10	Right	V3	-	I	II	-	0.17
5	Female	59	4	Left	V3	-	I	III	-	0.09
6	Female	67	2	Right	V3	RF	I	II	Herpes labialis	0.15
Mean		65.6	7.5							0.142

### Accuracy, Effectiveness, and Safety

Bone fiducials registration was successfully completed in all six patients, and the mean registration error was 0.14 mm. Twelve trajectories (trajectory A and B for each patient) were successfully reached without any block from the skull. The positions of cannulas were adequate, and pear-shaped balloons appeared in all instances after injection of 0.5–0.7 mL of contrast material.

All patients achieved immediate pain relief following PBC and were classified as having BNI Pain Intensity Score grade I. Five patients (83.3%) exhibited facial numbness and hypoesthesia, including four cases with BNI numbness score grade II and one with grade III. The sensory symptoms were transient and entirely resolved within 3 months following the operation. Transient masseter muscular weakness was encountered in one patient (16.7%) and entirely resolved within 2 months. No permanent or severe complications were encountered in this group of patients, including neural or vascular neural injuries, oral hematomas, diminished corneal reflexes, or infections. According to the last follow-up, no pain recurrence was reported within the relatively short follow-up period.

The average distance of the puncture route from the skin to the foramen ovale (trajectory A) for the six patients was 73.9 mm (range, 70.8–79.1 mm). Since trajectory B required pushing against the skin, we did not record the distance of this puncture route. To calculate the positional relationship between the two puncture routes at different angles, we calculated the position of the two puncture routes at the arc-angle and the ring-angle on the anterior commissure-posterior commissure plane, and then determined their positional relationship at different angles. Compared with trajectory A, trajectory B moved laterally by an average of 3.2 degrees at the arc-angle and moved downward by an average of 5.82 degrees at the ring-angle. The distance and the angles of the two trajectories are shown in Table 2.

**Table 2:** Length and angles for the trajectories A and B.

**Patient**	**Direction**	**Trajectory A(degrees)**	**Trajectory B(degrees)**	**Arc adjustment(degrees)**	**Ring adjustment(degrees)**	**Length of trajectory A (mm)**
1	Arc	104.4	105.8	−1.4		73.8
	Ring	320.7	313.9		6.8	
2	Arc	72.5	71.3	1.2		74.5
	Ring	319.3	312.3		7	
3	Arc	75.5	73.4	2.1		73.2
	Ring	309.9	305.3		4.6	
4	Arc	73.7	69.8	3.9		79.1
	Ring	310.6	304.6		6	
5	Arc	104.5	107.2	−2.7		72.2
	Ring	312.7	307.8		4.9	
6	Arc	69.8	61.5	8.3		70.8
	Ring	311.2	305.6		5.6	
mean	Arc	16.237	19.5	3.27		73.9
	Ring	314.07	308.25		5.82	

## Discussion

MVD is recommended as the first option for TG treatment because it has a considerably higher rate of initial pain-free outcomes and a lower rate of long-term recurrence than other percutaneous procedures. However, PBC offers the advantages of being minimally invasive, requiring a shorter operation time and being less frequently associated with serious complications. These features make PBC an attractive choice for TN treatment, especially among elderly and infirm patients (16–18).

Although rare, serious complications related to needle misplacement such as carotid cavernous fistulae and external carotid artery system fistulae, subarachnoidal hemorrhage, intraparenchymal hematoma, and blindness have been reported for PBC (9, 19, 20). Facial numbness, hypoesthesia, and mastication weakness were the most common postoperative complications. They might have been caused by repeat punctures or long compression times, and were usually resolved within weeks or a few months after surgery at the latest (16, 21). An accurate surgical puncture may therefore improve the surgical outcome and reduce complications (21).

Based on our experience, the PBC surgical technique is focused on two aspects: foramen ovale puncture and Meckel's cave puncture. PBC consists of three sequential stages, each with specific steps: (1) foramen ovale insertion, (2) Meckel's cave cannulation, and (3) compression of the retroganglionic rootlets.

Several authors have introduced various methods to improve the success rate and the speed of foramen ovale puncture. At present, most surgical units perform the surgery using C-arm fluoroscopy, but observation or identification of the foramen ovale using this method is still a challenge. The difficulty in the identification of the foramen ovale is even more pronounced in patients with anatomic variations at the skull base. Therefore, some authors have proposed the use intraoperative 3D CT-guided puncture to improve the success rate of foramen ovale puncture, especially in patients with a narrow foramen (12, 22, 23). This method can improve the accuracy of the puncture, but multiple puncture attempts and adjustments are still required, and the patient needs to undergo multiple CT scans. Neuronavigation was also used to achieve accuracy during puncturing, but this approach requires the surgeon to monitor real-time videos, and registration duration increases while accuracy decreases when compared with the use of robots. A personalized 3D-printed jig plate could be an effective tool for rapid foramen ovale puncture, with a duration of around 1 min (11). However, the angle and depth of the puncture needle still need to be manually adjusted and controlled to access Meckel's cave.

Meckel's cave is located in most cases above the foramen ovale in the line that connects the puncture entry point in the facial region and the foramen ovale. The tail end of the puncture needle needs to be pressed downwards and the needle further advanced by ~1 cm to access Meckel's cave (24). This represents a second challenge for the surgeon, since mistakes can lead to puncture failure or damage to the nerves, blood vessels, or the cavernous sinus. Excessive penetration of the puncture needle might also cause damages to the brain stem, especially in patients with a broad foramen ovale. None of the previously mentioned approaches allows for accurate control of the depth and direction of the puncture needle. Therefore, the surgeon has to rely on surgical experience and anatomical knowledge to tackle these challenges, as well as attempt the puncturing multiple times (13).

Our study used robot-assisted technology to navigate the challenges described above. This technology was confirmed to be safe and effective when applied in the context of stereoelectroencephalography (SEEG) implantation, deep brain stimulation, and other neurosurgical operations. Its planning station can design the puncture trajectory preoperatively to accurately locate and puncture the foramen ovale and Meckel's cave, while its robotic arm can accurately control the depth and direction of the puncture needle.

We designed trajectories A and B for foramen ovale puncture and Meckel's cave puncture, respectively. Trajectory B with the target point set on Meckel's cave was created because the tip of the puncture needle would touch the temporal bone instead of reaching Meckel's cave if trajectory A was used for this purpose. Previous studies suggested that the tip of the puncture needle needs to be adjusted when advanced from the foramen ovale to Meckel's cave, but no specific data was provided (24, 25). Our study shows that the tail of the puncture needle had to be moved laterally by an average of 3.2 degrees at the arc-angle and moved downward by an average of 5.82 degrees at the ring-angle after the needle has reached the foramen ovale. When accessing Meckel's cave, the puncture needle had to be moved laterally to the medial line by 19.5 degrees at the arc-angle and by 308.25 degrees at the ring-angle. Despite the small sample size, these findings might already provide some guidance to surgeons attempting the procedure.

In this study, the target points were successfully accessed after adjusting once in all six patients, and no other adjustments were performed. Previous studies did not mention the number of puncture attempts required when the puncture needle was advanced from the foramen ovale to Meckel's cave. Our approach minimizes the risk of potential complications triggered by unsuccessful puncture attempts and reduces the patient's exposure time to radiation. Trajectory B could be adopted directly, but since the entry point is too low, there is a risk of penetration of the oral cavity by the needle.

The registration error was only 0.14 mm during the operation, which provides a firm guarantee for accurate puncture. Needle entry was not blocked by the bone in any puncture route, which suggests that the robotic arm followed the exact planned puncture route during the operation. Since intraoperative CT was not used, the accuracy in target point puncture cannot be verified. However, given the accuracy of previous electrode implantations, the probability of puncture needle misplacement is very low.

### Learning Curve

Although experienced neurosurgeons can easily perform PBC with good results and low chances of complications, the technique has a steep learning curve. Intraoperative CT, neuronavigation, and the use of a 3D-printed jig plate in the initial stages of learning have been reported to be useful during training (11, 13). In our study, the surgeon had previously performed SEEG and deep brain stimulation (DBS) electrode implantation in thousands of cases, but he had no previous experience performing PBC. We successfully achieved effective compression thanks to the accuracy provided by the robotic equipment and the visualization of the surgical trajectories. Our approach can therefore help many surgeons who are not familiar with PBC. The visualized design of the puncture routes, in particular, can improve the surgeon's anatomical understanding of the foramen ovale and Meckel's cave.

### Limitations

Some limitations of the present study are the small sample size and the relatively short duration of the follow-up periods. We are collecting more cases at present. Robot-assisted surgeries require longer preparation time and fixation of the patient's head with a skull clamp. To reduce scalp injuries caused by bone screws, we are attempting the use of structured light 3D scanning for registration. In addition, we are also attempting to reduce the duration of the surgery by using a single puncture route.

## Conclusions

PBC is an effective and minimally invasive treatment for TG. This study proposes a novel surgical approach for PBC. Despite requiring a longer time for preoperative preparation than previous approaches involving direct puncture, our approach provides a high degree of accuracy and safety. Robot-assisted PBC can also shorten the learning curve substantially, especially for surgeons unfamiliar with PBC. Robots have been widely deployed for SEEG and DBS electrode implantations; likewise, robot-assisted surgical approaches should be further developed and widely adopted in PBC.

## Data Availability Statement

The original contributions presented in the study are included in the article/supplementary material, further inquiries can be directed to the corresponding author.

## Ethics Statement

The studies involving human participants were reviewed and approved by Ruijin Hospital Luwan Branch Ethics Committee, Shanghai JiaoTong University School of Medicine. The patients/participants provided their written informed consent to participate in this study.

## Author Contributions

QL: conceptualization, methodology, and funding acquisition. JW: writing - original draft and investigation. CW: software and investigation. WenzeC: writing - original draft and software. WenzhC: data curation and writing - original draft. XY: data curation and writing - original draft. ZM: data curation. CZ: writing - review & editing. JX: supervision and writing - review & editing. All authors contributed to the article and approved the submitted version.

## Funding

The research and publication of this article was funded by the Shanghai Jiao Tong University Fund for Interdisciplinary Research for Medical Applications [YG2021QN30].

## Conflict of Interest

The authors declare that the research was conducted in the absence of any commercial or financial relationships that could be construed as a potential conflict of interest.

## Publisher's Note

All claims expressed in this article are solely those of the authors and do not necessarily represent those of their affiliated organizations, or those of the publisher, the editors and the reviewers. Any product that may be evaluated in this article, or claim that may be made by its manufacturer, is not guaranteed or endorsed by the publisher.

## Abbreviations

BNI: Barrow Neurological Institute
CT: Computed tomography
DBS: Deep brain stimulation
GR: Glycerol rhizolysis
MVD: Microvascular decompression
PBC: Percutaneous balloon compression
RF: Radiofrequency ablation
SEEG: Stereoelectroencephalography
TG: Trigeminal neuralgia.
